# Rapid Spur Gear Profile Inspection Using Chromatic Confocal Sensors

**DOI:** 10.3390/s26030874

**Published:** 2026-01-28

**Authors:** Bo-Huang Chang, Tsung-Han Wu, Wei-Chieh Chang, Chung-Ping Chiang, Wei-Hua Chieng

**Affiliations:** 1 Department of Mechanical Engineering, College of Engineering, National Yang Ming Chiao Tung University, Hsinchu 30010, Taiwan; cbh.en12@nycu.edu.tw; 2 Mechanical and Mechatronics Systems Research Laboratories, Industrial Technology Research Institute, Hsinchu 31040, Taiwan; itria40195@itri.org.tw (T.-H.W.);

**Keywords:** chromatic confocal sensors, involute gear profile, non-contact measurement, in situ measurement, gear inspection

## Abstract

**Highlights:**

**What are the main findings?**

- A novel method for in situ inspection of gear profiles using a Chromatic Confocal Sensor (CHCS) is proposed.
- The verification and validation of this gear profile inspection method have been performed via software simulations and experiments.

**What are the implications of the main findings?**

- Less time-consuming, high throughput gear inspection.
- Reliable quality control for gear inspection.

**Abstract:**

Gears, as critical power-transmission components in most power equipment, have a particularly urgent need for in situ inspection systems. Traditional gear inspection methods rely on contact inspection instruments, which are not only time-consuming, but also potentially damage the gear surface due to contact. This study delves into the detection requirements in the gear manufacturing process and establishes a rapid, non-contact detection mechanism and model using a CHCS. This model employs a CHCS to achieve high-speed, non-contact measurement on various surfaces with extremely high accuracy, enabling real-time monitoring of production process details, thereby improving production efficiency and ensuring product quality. Through actual inspection and comparison with a standard involute spur gear tooth profile model, this study implements a complete inspection system in a prototype. The results of gear inspection using a CHCS with an accuracy of 1 μm showed that the interquartile range of qualified gears under test (GUTs) was within 2.5 μm, and the beard line value was within 10 μm. The experiment demonstrated a layout equipped with a CHCS where the rotating axis represents the hobbing machine spindle. This method can be completed without moving the gear, enabling subsequent finishing processes.

## 1. Introduction

According to the study by Shao et al. (2022), the existing interpretation of the Intergovernmental Panel on Climate Change (IPCC) Sixth Assessment Report (AR6) suggests that, in order to limit global warming to 1.5 °C, global industrial emissions may need to be reduced by about 50–100% from the 2019 levels by 2050 [1]. Promoting the net-zero transformation of industries and related measures is imperative. Against this backdrop, green energy industries, electric vehicle industries, and other gear-related industries are booming. In the gear-fabrication process, traditional gear precision testing methods, whether contact gear testers, three-dimensional measuring instruments, or gear meshing test methods, all require transferring gears from the production line to the laboratory. In addition, the gears being tested cannot be repaired online, and once they are judged to be unqualified parts, they can only be scrapped or recycled. Traditional testing methods are time-consuming and labor-intensive, and will increase carbon pollution.

Recently, an advanced multi-camera system based on stereo vision [2] has been developed for the automatic, high-precision, and efficient measurement of the three-dimensional profile of pipes. The average deviations of both radial and axial offsets in this system are within a reference value of 62 μm, with a maximum standard deviation of 25 μm. Furthermore, a series of simplified solvers for calculating the six-DOF relative pose of multi-camera systems using beam constraints [3] have also been developed. These solvers can reduce the solution space and develop more stable solvers. Rotation errors can be less than 0.2 degrees.

Traditional gear testing methods fall into two categories: measurements emphasizing quantitative analysis and testing emphasizing qualitative analysis [4]. Quantitative measurements are usually performed using a Coordinate-Measuring Machine (CMM) [5] with specialized software or a professional Gear-Measuring Instrument (GMI) [6], following national and international gear standards, such as ISO/IEC, GB/T, DIN, and AGMA [7,8,9,10]. Qualitative testing is performed using a Single Flank Test (SFT) on a single-tooth meshing machine [11] or a Double Flank Test (DFT) on a double-tooth meshing machine [12,13]. Testing also needs to comply with the aforementioned national and international standards. SFT offers higher accuracy than DFT but is slower, whereas DFT cannot detect deviations such as pitch circle errors or tooth profile errors [14]. CMMs and GMIs are large, high-precision, and expensive, requiring careful setup. Both CMM and GMI need to be installed in large, positive-pressure laboratories with controllable temperature and humidity to minimize environmental sources of error [15]. As a result, these instruments are typically located in professional laboratories. In addition to the high cost of laboratory maintenance, transporting gear samples from the factory to the laboratory also incurs considerable expense. SFT and DFT require the manufacture and calibration of standard gears for meshing tests. Moreover, these tests are often associated with gear wear, breakage, noise, and even machine failures or gear damage during meshing. In addition, SFT and DFT are expensive to acquire and maintain.

All of the gear testing instruments and methods mentioned above require probes or gear contact to achieve testing. To avoid wear and collision, non-contact optical testing methods are used. The principle is to use Charge-Coupled Devices (CCDs) to capture light signals. Common light sources include visible light sources and various colored laser light sources. Among them, lasers can use their interference principle to perform nanometer-level precision measurements [16]. However, when using lasers in combination with CMMs to measure surface contours, the laser itself is greatly affected by reflection and transparent surfaces [17]. Although the measurement resolution can be improved to 5 μm with the help of light-shielding technology [18], it is less sensitive to the small irregular contours of small gear surfaces [19] and cannot achieve the required standard measurement uncertainty [20]. Since gear accuracy and measurement uncertainty will increase with the increase in gear size, it is applied to the measurement of large gears [21]. Because lasers cannot be applied to reflective and transparent materials, the task of measuring reflective and transparent materials is taken over by CHCS [22]. CHCS is an advanced, non-contact optical distance-measurement technology that achieves high-speed and high-precision axial distance measurement through innovative design. CHCS technology overcomes the limitations of traditional optical measurement methods in terms of speed and surface adaptability, making it an ideal choice for high-precision industrial inspection. The core principle of the CHCS system is to utilize the principles of confocal and dispersion. This system uses a dispersive objective lens. When light emitted from a broadband light source (such as a high-intensity light-emitting diode, LED) enters this objective lens, the chromatic aberration phenomenon is intentionally amplified, and light of different wavelengths will be focused at different positions along the optical axis. This establishes a continuous spectral focal line on the optical axis, which uniquely encodes the spatial distance and the wavelength of light. The position of the object under test in the axial direction corresponds to the reflected light of different wavelengths [23], thereby achieving precise measurement with a maximum linear error of only 0.7 μm [24].

Therefore, this study proposes a gear inspection method that can be installed on the production line. It utilizes a non-contact CHCS to replace the ruby contact probes used in traditional CMM and GMI methods. This avoids the possibility of collisions, space constraints, and high costs associated with on-machine contact inspections. It also provides a faster inspection rate to verify the processing results by inspecting the entire tooth profile. This study uses a standard spur gear as the inspection sample and compares and verifies the inspection results using a theoretical standard spur gear tooth profile model. The reliability and accuracy of the method are then verified through multiple reproducible standard spur gear inspection experiments. The non-contact gear inspection method in this study is based on the method proposed by Marc Pillarz [25,26], further refining it into a more comprehensive and rapid CHCS tooth profile inspection system. This system offers advantages such as comprehensiveness, speed, ease of installation, small installation space, no collision wear, and lower cost. The chromatic aberration conjugate focus sensor manufactured by PRECITEC uses a high-performance optical lens to focus different colors of light at different distances along the optical axis [27]. The spot size projected onto the gear profile is approximately 6 mm in the 40° direction. CHCS provides precise depth and thickness measurements, even on transparent or highly reflective materials.

Traditional inspection methods include CMMs and GMIs that measure involute profiles, helices, and pitch, and optical profile projectors that magnify the gear profile and compare it with a standard template. All of these traditional methods require transferring the GUT from the machine tool to the laboratory. In-line inspection methods include advanced multi-camera systems based on stereo vision (for 3D profile measurement) and line-scanning lasers, but by 2026, none of these methods will achieve an accuracy better than 2 μm. CHCS is an optical device that uses white and dispersed light to obtain distance by analyzing the reflectance spectrum and correlating it with a specific wavelength, achieving a vertical resolution of 30 nm. Under appropriate temperature control, the CHCS can achieve an accuracy better than 1 μm. However, laser scanning and image processing present problems when measuring concave profiles. Therefore, we recommend placing the CHCS at an offset position from the gear profile and attempting to focus the optics as vertically as possible on the surface.

This paper is organized into several sections, including the goal and method of our gear testing in the Section 1, the gear profile conventions and derivation for CHCS testing in the Section 2.2, the analysis method using the CHCS system in the Section 3, and the verification and validation of the method proposed in this paper in the Section 4.

## 2. Geometric Profile

### 2.1. Involute Line

The pitch circle radius of a gear is defined as follows.

(1) $$R_{p}=\frac{m\cdot N}{2}$$

where m denotes the module of the gear. N denotes the number of teeth of the gear. The tooth pitch is defined as follows.

(2) $$d_{p}=\pi m$$

The radius of base circle, $R_{ba}$, is a function of module, number of gear teeth, and the pressure angle as follows.

(3) $$R_{ba}=R_{p}\cdot cos\alpha$$

where α denotes the pressure angle of the gear mesh. The addendum circle radius, $R_{ad}$, is defined as follows.

(4) $$R_{ad}=R_{p}+m$$

The gear involute line of the tooth-right, $G_{q}$, in terms of parameter q is derived as follows.

(5) $$G_{q}=\left[\begin{array}{c} x_{q}\\ y_{q} \end{array}\right]=R_{ba}\left[\begin{array}{c} cos\left(q\gamma \right)+q\cdot \gamma \cdot sin\left(q\gamma \right)\\ sin\left(q\gamma \right)-q\cdot \gamma \cdot cos\left(q\gamma \right) \end{array}\right],q_{0}\leq q\leq q_{t}$$

where $\gamma =\pm \frac{\sqrt{{R_{ad}}^{2}-{R_{ba}}^{2}}}{Q\cdot R_{ba}}$

$q_{0}$ and $q_{t}$ are associated with the starting and final parameter q of the involute line, respectively. The signs ± correspond to the tooth-right and tooth-left involute lines, respectively. In terms of the gear rotation angle ϕ, we let $G_{\phi }$ denote the involute line position in Cartesian coordinate, which may also be written in terms of the polar coordinate parameters.

(6) $$G_{\phi }=\left[\begin{array}{c} x_{\phi }\\ y_{\phi } \end{array}\right]=R_{\phi }\left[\begin{array}{c} cos\phi \\ sin\phi \end{array}\right]$$

where $R_{\phi }=\sqrt{{x_{q}}^{2}+{y_{q}}^{2}}=R_{ba}\cdot \sqrt{1+{\left(q\cdot \gamma \right)}^{2}}$

The gear rotation angle ϕ for the involute line shall be applicable for all teeth, which may be redefined according to the tooth index n as follows.

(7) $$\phi \equiv \Phi \left(q\right)={tan}^{-1}\frac{y_{q}}{x_{q}}-{tan}^{-1}\frac{y_{q_{p}}}{y_{q_{p}}}+\phi_{p},\;q_{0}\leq q\leq q_{t}$$

where $\phi_{p}=\frac{360\cdot \left(n-0.75\right)}{N}$

$q_{p}\;$ is the parameter q where the involute line intersects the pitch circle.

(8) $$q_{p}=\frac{1}{\gamma }\sqrt{\frac{1}{{cos}^{2}\alpha }-1}$$

where α denotes the pressure angle, as defined in Equation (3). The gear top land radius, $R_{t}$, corresponding to $q_{t}$, which may be larger than the addendum circle in practice, is derived as follows.

(9) $$q_{t}=Q\sqrt{\frac{{R_{t}}^{2}-{R_{ba}}^{2}}{{R_{ad}}^{2}-{R_{ba}}^{2}}}$$

The standard value of addendum circle radius, $R_{de},$ and the associated parameter $q_{de}$ are formulated as follows.

(10) $$R_{de}=R_{ba}-1.25\cdot M$$

The addendum circle does not intersect the involute line; therefore, the gear angle $\phi_{de}$ is defined separately as $\phi_{de}$.

### 2.2. Gear Profile

The first tooth is the tooth that has the smallest ϕ, which indexed as tooth 1 and other teeth are indexed as 2, 3, 4… up to N in counterclockwise (CCW) direction. It is therefore possible to define half of the tooth pitch angle, $\phi_{1}$, for locating the center of the top land as follows.

(11) $$\phi_{1}=\frac{180^{{}^{\circ}}}{N}$$

The involute line is started from $q_{0}$ and the starting ϕ of the involute line is

(12) $$\phi_{0}=\Phi (q_{0})$$

The gear top land circle radius, $R_{t}$, corresponds to $q_{t}$ which is defined as follows.

(13) $$\phi_{t}=\Phi (q_{t})$$

The arc angle of the top land is expressed as follows.

(14) $$\phi_{tl}=\phi_{1}-2\cdot \left(\phi_{t}-\phi_{p}\right)$$

**Figure 1:**
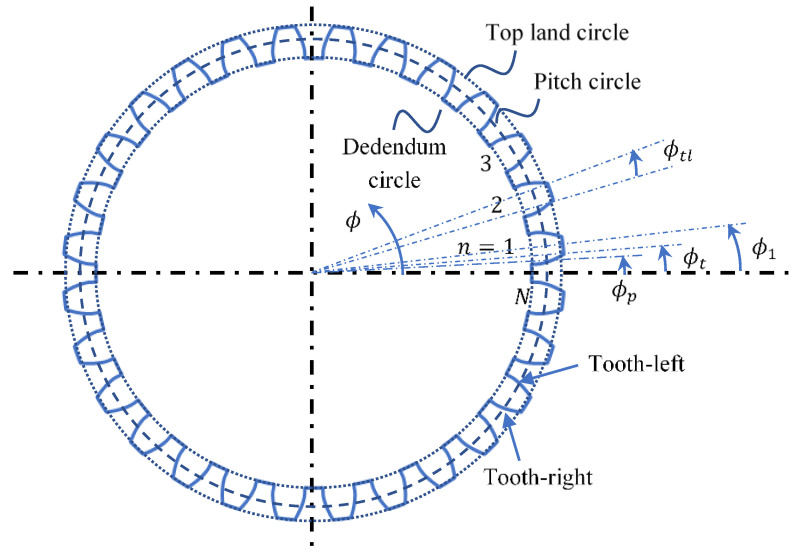
The spur-gear cross-section and definitions (dotted lines indicate the locations of corresponding angle definitions).

As shown in Figure 1, the tooth-left and tooth-right involute lines are distinguished by the sign in Equation (6); the tooth-left is the one with positive sign and the tooth-right is the one with negative sign.

Ignoring the top-land corner and dedendum cornering functions, we simplified the gear profile F(ϕ) using one of the half dedendum curve, tooth-right, top-land curve, tooth-right and the other half of the dedendum curve, which are expressed separately as follows.

(15) $$F(\phi )=\{\begin{array}{cc} B\left(R_{de},\;\phi \right), & \phi_{0}>\phi \geq 0\\ G_{\phi ,l}, & \phi_{t}>\phi \geq \phi_{0}\\ B\left(R_{t},\;\phi \right), & \phi_{t}+\phi_{tl}>\phi \geq \phi_{tl}\\ G_{\phi ,r}, & 2\phi_{t}+\phi_{tl}-\phi_{0}>\phi \geq \phi_{t}+\phi_{tl}\\ B\left(R_{de},\;\phi \right), & \frac{360}{N}>\phi \geq 2\phi_{t}+\phi_{tl}-\phi_{0} \end{array}$$

B(r, ϕ) is a circle function defined as follows.

(16) $$B\left(r,\;\phi \right)=r\left[\begin{array}{c} cos\phi \\ sin\phi \end{array}\right]$$

$G_{\phi ,l}$ and $G_{\phi ,r}$ denote the involute lines for tooth-right and tooth-right respectively. Since all curves are defined using the polar coordinate, the rotation of the gear profile by an angle θ can be simply expressed as follows.

(17) $$F(\phi +\theta )=\left[\begin{array}{cc} cos\theta & -sin\theta \\ sin\theta & cos\theta \end{array}\right]F(\phi )$$

An example of the standard spur gear specifications and the corresponding calculated results are shown in Table 1.

## 3. Chromatic Confocal Sensor (CHCS) Scan and Error Model

### 3.1. CHCS Scan Arrangement

CHCS is an advanced, non-contact optical distance measurement technology that achieves high-speed, high-precision axial distance measurement through innovative design. CHCS technology overcomes the limitations of traditional optical measurement methods in terms of speed and surface adaptability, making it an ideal choice for high-precision industrial inspection. The core of the CHCS system lies in its dispersive objective lens. When light emitted from a broadband light source (such as a high-intensity LED) enters this lens, due to the intentional amplification of chromatic aberration, light of different wavelengths is focused at different positions along the optical axis. This establishes a continuous spectral focal line along the optical axis, uniquely color-encoding the spatial distance with the wavelength of light. CHCS relies on the technologies of focusing and reflection, confocal filtering, and spectral decoding. When the spectral focal line is projected onto the surface of the GUT, this portion of the light is reflected back to the sensor system by the surface of the GUT. In the return optical path, the reflected light must pass through a precisely aligned confocal pinhole with confocal filtering. The light passing through the pinhole enters the spectrometer. Spectral decoding analyzes the received light signal and accurately detects the wavelength with the highest intensity.

**Figure 2:**
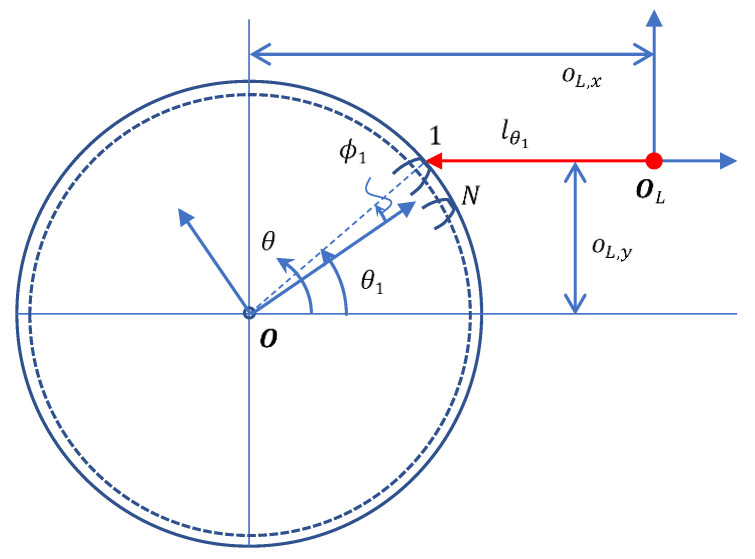
The CHCS scan definition.

The CHCS scan instrument is shown in Figure 2. Let $O_{L}$ denote the origin of the CHCS instrument coordinate system, which is controlled by a high-precision servo platform.

(18) $$O_{L}=\left[\begin{array}{c} o_{L,x}\\ o_{L,y} \end{array}\right]$$

A positive value of $o_{L,y}$ indicates the y-offset of the CHCS head, which allows for more CHCS points on the tooth-right of the gear profile. A motor is used to rotate the gear at a constant speed that results in the CHCS point traversing on the gear profile in the direction opposite to the motor rotation. There may be a range of the same minimum value of CHCS readout $l_{\theta }$ corresponding to the tooth top land; the motor angle $\theta_{1}$ is determined by the center location of the top land of the first tooth.

(19) $$\theta_{1}={sin}^{-1}\left(\frac{o_{L,y}}{R_{t}}\right)$$

In order to trace the involute lines, there are two sets of motor rotation, as follows.

**Figure 3:**
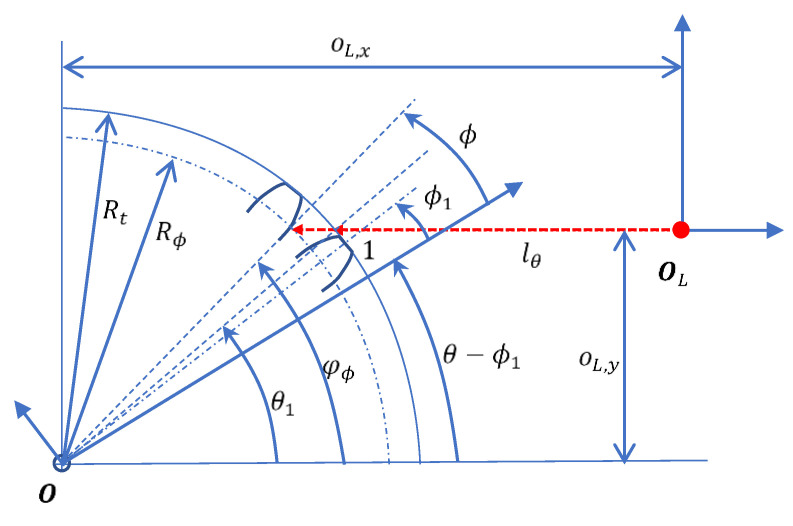
Location of the starting tooth of the gear and definitions.

1. Tooth-right involute line tracing: The y-offset of the CHCS $\;o_{L,y}$ is positive and the motor causes the gear to rotate in the clockwise (CW) direction. The CHCS readout starts from tooth 2, then proceeds to tooth N, N−1, and so on, as shown in Figure 3.
2. Tooth-left involute line tracing: The y-offset of the CHCS $\;o_{L,y}$ is negative and the motor causes the gear to rotate in the CCW direction The CHCS readout starts from tooth 1, then proceeds to tooth 2, 3, and so on.

The gear profile angle ϕ is derived from the motor rotation angle as follows.

(20) $$\theta =\phi_{1}+\varphi_{\phi }-\phi$$

where $\varphi_{\phi }$ denotes the CHCS spotting angle, which is equal to $\theta_{1}$ in the starting position when $\phi =\phi_{1}$. When the CHCS intersects the involute line the motor rotation, the corresponding CHCS spotting angle $\varphi_{\phi }$ deviates from the starting spotting angle $\theta_{1}$ due to the radius $R_{\phi }$ being different from the top land radius $R_{t}$, which is derived as follows.

(21) $$\varphi_{\phi }\equiv \varphi \left(\phi \right)={sin}^{-1}\left(\frac{o_{L,y}}{R_{\phi }}\right)$$

The CHCS scan result is correlated to the gear profile F in the direction opposite to the motor rotation, as follows.

(22) $$\left[\begin{array}{c} l_{\theta }\\ 0 \end{array}\right]=O_{L}-F\left(\phi \right)$$

It should be noted that $\varphi (\phi_{1})=\theta_{1}$. According to Equations (15)–(17), the CHCS readout can be derived as follows.

(23) $$l_{\theta }=o_{L,x}-R_{\phi }\cdot cos(\varphi_{\phi })$$

**Figure 4:**
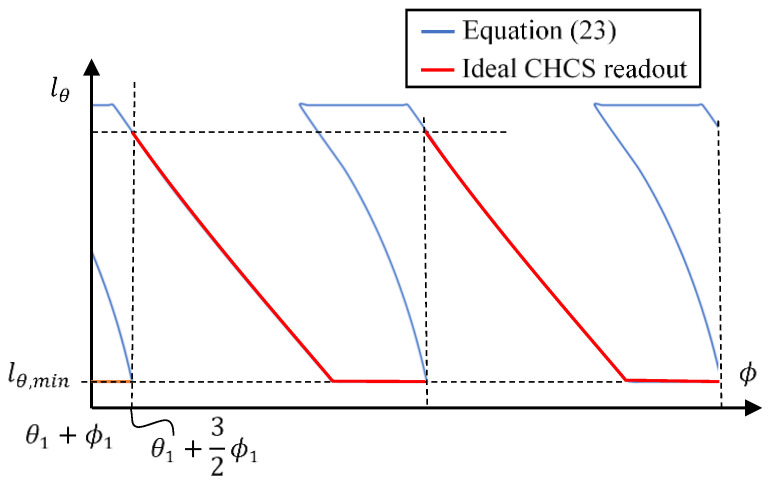
The ideal CHCS readout derivation.

For example, for the tooth-left of the gear profile scan, we may set $o_{L,y}=-18.658$ and according to the parameters specified in Table 1 and Equation (20) to obtain $\theta_{1}={-39.4708}^{{}^{\circ}}$. The CHCS readout $l_{\theta }$ associated with $o_{L,x}=23.509$ yields a violin-like plot, as shown in Figure 4. The gear profile yields multiple values on the same ϕ, which cannot physically occur because the CHCS will be blocked by the top land of the adjacent tooth when the CHCS is approaching the dedendum of the scanning tooth. Derived from Equation (21), CHCS non-blockage occurs when the following condition is reached.

(24) $$\phi -\left(\phi_{1}+\frac{\phi_{tl}}{2}\right)+\theta_{1}\geq {sin}^{-1}\left(\frac{o_{L,y}}{R_{\phi }}\right)$$

**Figure 5:**
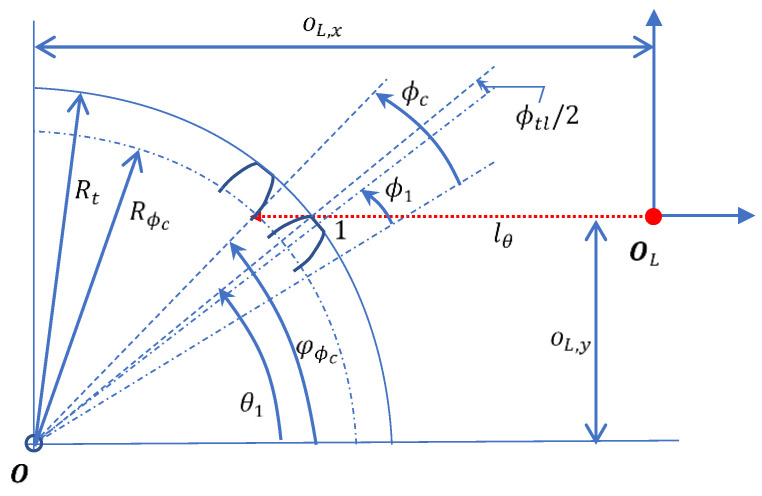
Motor rotation angle vs. gear profile angle and definitions.

The critical condition for the radius and angle of gear profile as shown in Figure 5 is derived as follows.

(25) $$R_{\phi_{c}}=\frac{o_{L,y}}{sin\left(\phi_{c}-\left(\phi_{1}+\frac{\phi_{tl}}{2}\right)+\theta_{1}\right)}$$

According to the equation, we can reduce the displacement $o_{L,y}$ to scan the proper portion of the dedendum profile.

### 3.2. Error Due to Gear Center-Offset

**Figure 6:**
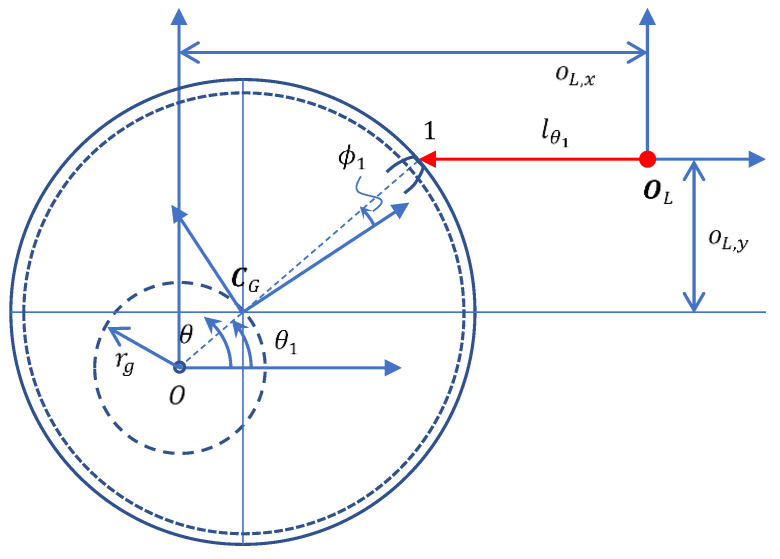
The gear center-offset definition.

With the CHCS instrument as shown in Figure 2, the gear may be installed in the off-line test inaccurately, which results a tiny center-offset, $r_{g}$, from the motor center O, as shown in Figure 6. A specific location where the gear is rotated to an angle, $\theta_{1}$, is where $l_{\theta_{1}}$ is the minimum value among all teeth via the center offset. There may be a range of similar minimum values of $l_{\theta }$ corresponding to the same tooth top; the angle $\theta_{1}$ is calculated for the center the tooth top land circle from the range. The CHCS readout is reformulated for the tiny center-offset, as follows.

(26) $$\left[\begin{array}{c} {l^{\prime }}_{\theta }\\ 0 \end{array}\right]=O_{L}-F^{\prime }\left(\theta -\theta_{1}+\phi_{1}\right)$$

where $F^{\prime }\left(\theta -\theta_{1}+\phi_{1}\right)=F\left(\theta -\theta_{1}+\phi_{1}\right)+\left[\begin{array}{c} r_{g}cos\phi_{1}\\ r_{g}sin\phi_{1} \end{array}\right]$

The equation above can only hold for tiny gear center offsets in order to ensure that the gear angle $\phi_{1}$ is still corresponding to the motor rotation angle $\theta_{1}\;$, i.e., $\varphi^{\prime }(\phi_{1})=\theta_{1}$. The angle of ${\varphi^{\prime }}_{\phi }$ is affected by the center offset, as follows.

(27) $${\varphi^{\prime }}_{\phi }={sin}^{-1}\left(\frac{o_{L,y}-r_{g}sin\theta }{R_{\phi }}\right)$$

According to Equation (26), the CHCS readout can be derived when ignoring the CHCS direction being subjected to some tiny rotation relative to the gear frame, as follows.

(28) $${l^{\prime }}_{\theta }=o_{L,x}-r_{g}cos\theta -R_{\phi }\cdot cos({\varphi^{\prime }}_{\phi })$$

The gear center offset, $r_{g},$ can be calculated by comparing the ${l^{\prime }}_{\theta_{1}}$ value that yields the largest value, which can be obtained as follows.

(29) $$r_{g}=\frac{1}{2}\left({l^{\prime }}_{\theta_{1}+180^{{}^{\circ}}}-{l^{\prime }}_{\theta_{1}}\right)\cdot sin\theta_{1}$$

The distance $o_{L,x}$ is calculated from the gear top land radius, $R_{t}$, the CHCS range data, ${l^{\prime }}_{\theta_{1}}$, and the gear center offset, as follows.

(30) $$o_{L,x}=\left(R_{t}+r_{g}\right)\cdot cos\theta_{1}+{l^{\prime }}_{\theta_{1}}$$

**Figure 7:**
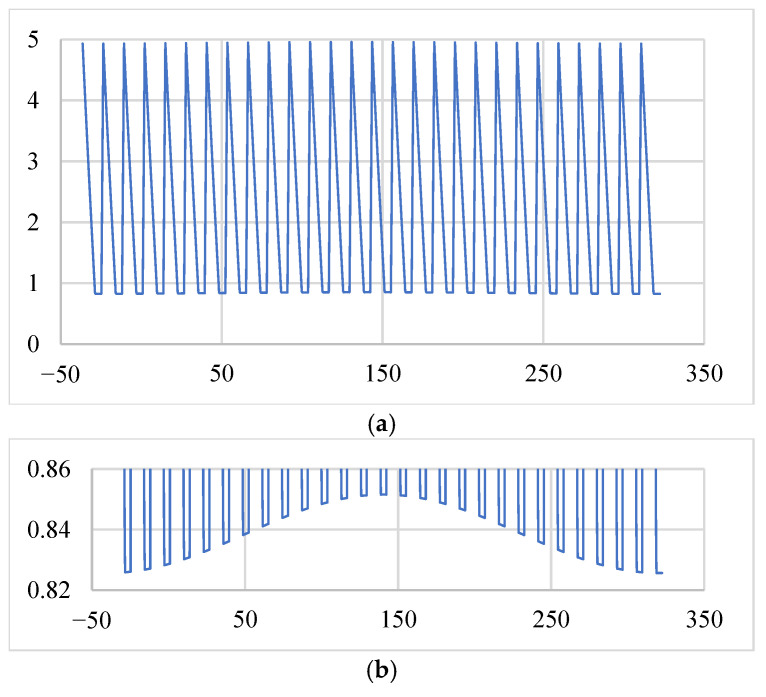
CHCS output due to the gear center-offset with (**a**) the effect of bottom shift and (**b**) a zoomed-in view.

For example, with $r_{g}=0.01$ and $\theta_{1}={-39.4708}^{{}^{\circ}}$, we calculated $o_{L,x}=23.509$ from the parameter $R_{t}$ listed in Table 1. The ideal CHCS readout under the parameters $o_{L,x}=23.509$ and $o_{L,y}=-18.658$ for the gear specified in Table 1 is shown in Figure 7a. Figure 7b shows a zoomed-in view of Figure 7a in the vicinity of the gear top-land to demonstrate the effect of gear center-offset to the CHCS readout.

## 4. Experiment and Error Analysis

Section 4 consists of four subsections, including: 4.1 Master Gear Test, used to analyze the error range of the main gear under CHCS measurement; 4.2 Gear Center Offset, used to extract the residual measurement error under the influence of gear center offset; 4.3 Accuracy Tolerance Specification of the GUT, used to construct the qualified/standard transmission error for gear inspection and based on the simulation of error sources to generate different gear profile templates for comparison with the master gear (these error sources are according to the gear standards (DIN, ISO, and AGMA)); and 4.4 Used Gear Inspection, to discover actual errors that may be caused by wear of the standard gear during transmission operation. The CHCS and its AD/DA processor are made by Precitec GmbH & Co. KG in Gaggenau, Germany.

### 4.1. Master Gear Test

**Figure 8:**
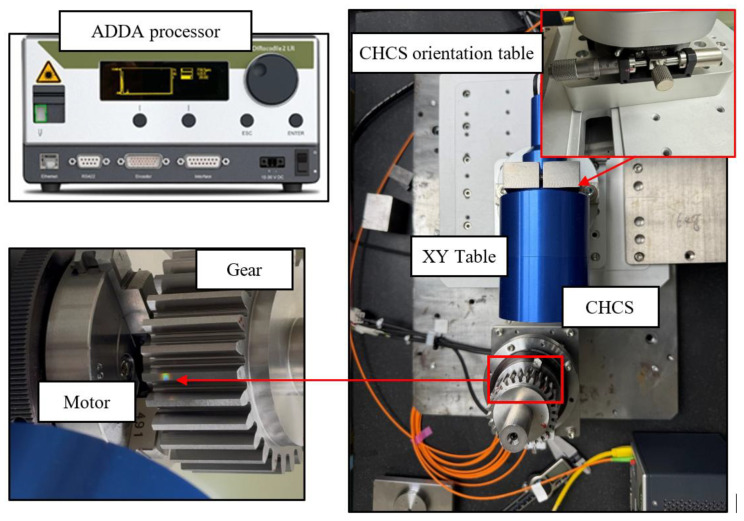
Experimental layout.

The experiment setup as shown in Figure 8 is equipped with a CHCS scan device with light spot diameter 6 mm, an AD/DA processor (CHRocodile 2S), a home-made precision XY table and its servo-control system, a CHCS orientation table, a home-made motor and its servo-control system to rotate the gear, and a temperature/humidity sensor. The AD/DA sampling frequency for the CHCS readout is 10 Hz when the motor rotation speed is $2^{{}^{\circ}}$/s; the testing time excluding the mounting procedures of the gear on the motor axis is then calculated to be within 6 min. The testing time for the gear profile reading is much faster than that when operated using the conventional CMM machine, which typically requires hours of testing time. In this paper, the parameters of the master gear are listed in Table 1. During the left and right tooth scans, the XY precision table has an x-direction offset of $o_{L,x}=23.509\;mm$ and a y-direction offset of $o_{L,y}=\pm 18.658mm$, corresponding to the left and right scans, respectively. The distances are calibrated according to the motor axle using the CMM machine and other calibration procedures for the CHCS calibration. The CHCS fabricated by PRECITEC uses high-performance optical lenses to focus white light at different distances along the optical axis, and not on a single point [27]. The features of the CHCS include measurement being possible on any kind of material, high slope acceptance and high numerical aperture that allow up to 45° on reflective surfaces and 80° on diffusive surfaces, high *Z*-axis resolution and accuracy, avoiding the shadowing effect, and a small and constant spot size with high lateral resolution. As shown in Figure 8, the spot size of the white light CHCS projecting on the gear profile is around 6 mm in the 40° direction. The CHCS orientation table is for the micro-adjustment of the CHCS heading direction.

**Figure 9:**
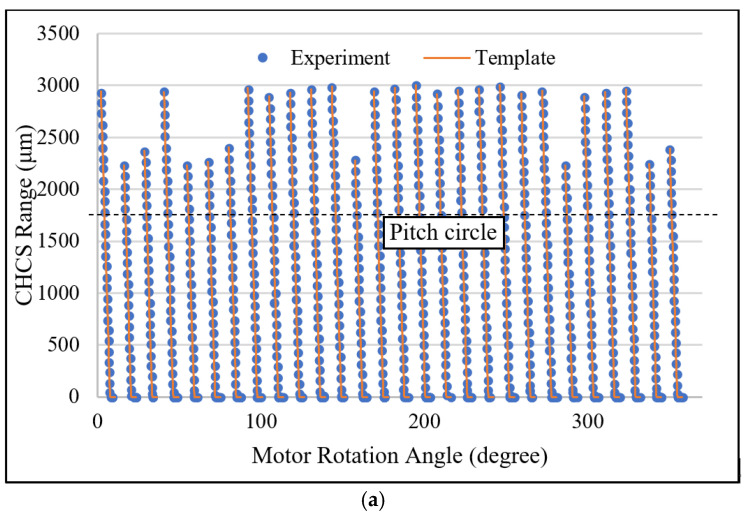

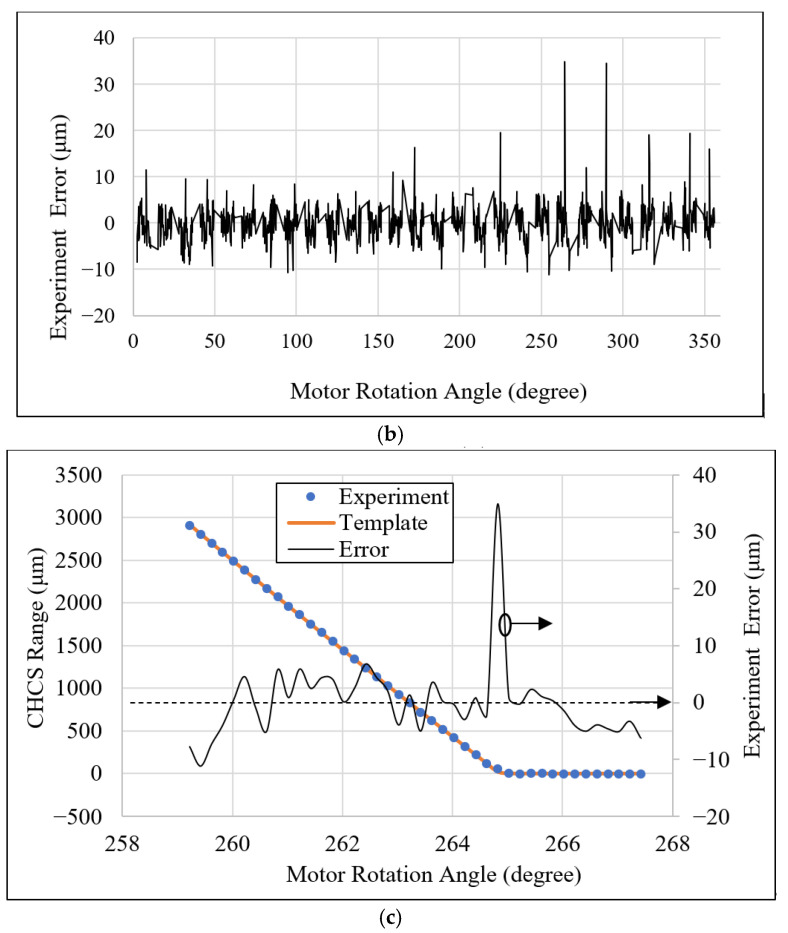
Tooth-left CHCS scan: (**a**) all-teeth comparison, (**b**) experiment error plot, and (**c**) tooth comparison.

The result of the tooth-left scan is shown in Figure 9. A gear with Level-4 standard grinding precision is mounted to the motor axle. The CHCS range distance from the pitch circle to the top-land of the gear is calculated from Equation (23) to be 1765 μm. In Figure 9a, we moved the bottom of each tooth to align with the individual template tooth at the top-land using the least-square method. The template gear profile under the CHCS scan is calculated from Equation (23) without considering the gear-center offset; therefore, the gear-center offset is left to study later. It is observed that all teeth are scanned well from the top-land to the pitch circle, when some of the teeth are scanned more toward the dedendum portion than others. The CHCS point mapping between the motor angle to the gear angles is determined according to Equations (20) and (21). The experiment errors of the tooth-left profile are calculated between the CHCS readout and the template discrete position according to the motor rotation angle reading θ from the motor controller. In the tooth-left scan, we set $o_{L,y}=-18.658\;mm$. The experiment error is depicted in Figure 9b. The maximum error is around 35 μm, which is selected for the detailed inspection of the error sources. In Figure 9c, the secondary Y axis shows the experiment error scale and indicates that the maximum error is due to the round corner of the top-land when the corner is precisely spotted by the CHCS. A large error may also occur at the dedendum area when the CHCS loses accuracy due to its spot size; it can be observed that there are many losses of data in the range between the pitch circle and the dedendum, as shown in Figure 9a. This may also due to the gear-center offset that causes the average line of tooth profile to be away from the zero-error line indicated in Figure 9c.

**Figure 10:**
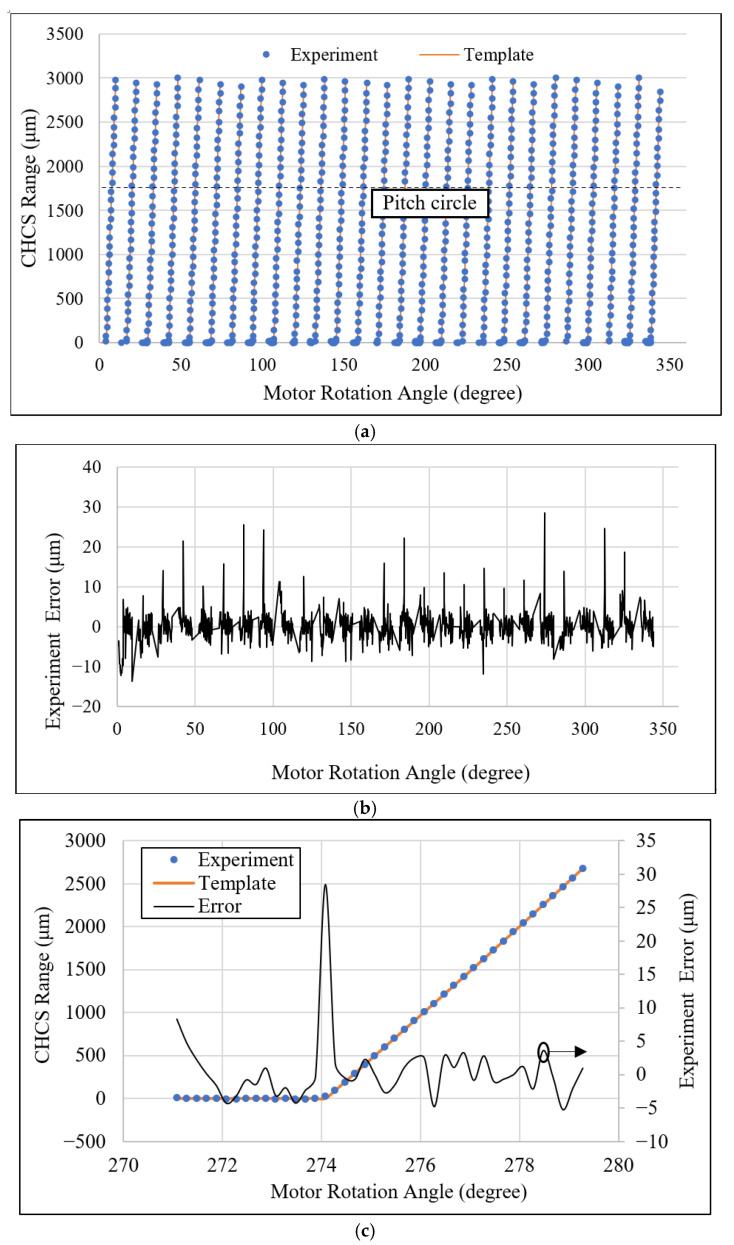
Tooth-right CHCS scan: (**a**) all-teeth comparison, (**b**) experiment error plot, and (**c**) tooth comparison.

The same CHCS scan procedure was applied to the tooth-right scan. In the experiment, we set $o_{L,y}=18.658\;mm$. The result is shown in Figure 10a. It may have been due to the clean surface condition that the tooth-right profile was scanned more toward the dedendum area. The experiment error shown in Figure 10b indicates that more gear top-land corners had been scanned; thus, many large errors were shown. When we examine the comparison of the teeth exerting more error in detail, the maximum error again occurs at the gear top-land rounding, as shown in Figure 10c. But, this time, significant error is also shown on the top-land, where the CHCS failed to yield a readout. This is due again to the CHCS spot size affecting the reading precision.

**Figure 11:**
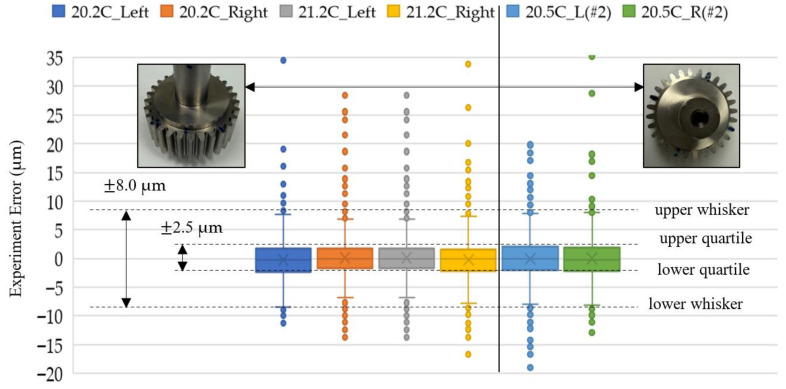
Box-and-whisker plot for experiment error analysis.

We repeated the experiment the second time on a different day. The box-and-whisker plot of the experimental error was obtained using two master gears and tested at different times to yield more robust repeatability and reproducibility statistics. The experiment error in Figure 9b and Figure 10b is fed into the analysis using Microsoft Excel software. Six sets of experiments represent the tooth-left and tooth-right scans with different temperatures, which are 20.2 °C, 21.2 °C and 20.5 °C. As shown in Figure 11,the box-and-whisker plot results show that all experiments with different directions of motor rotation and temperatures have their upper and lower quartiles within the bounds of ±2.5 μm. The upper and lower whiskers are within the bounds of ±8.0 μm. The outliers, as stated before, are due to the round corner of the gear top-land. Regarding the motor rotation and temperature difference effects, there is no significant difference among all of them. The humidity at 20.2 °C was 60.4%RH, that at 21.2 °C was 60.2%RH, and that at 20.5 °C was 59.8%RH. From the repeatability and reproducibility statistics, we may conclude that the box-and-whisker plot can be used in the rapid testing of the gear production by checking the quartile and whisker results of the gear under test.

### 4.2. Gear-Center Offset

**Figure 12:**
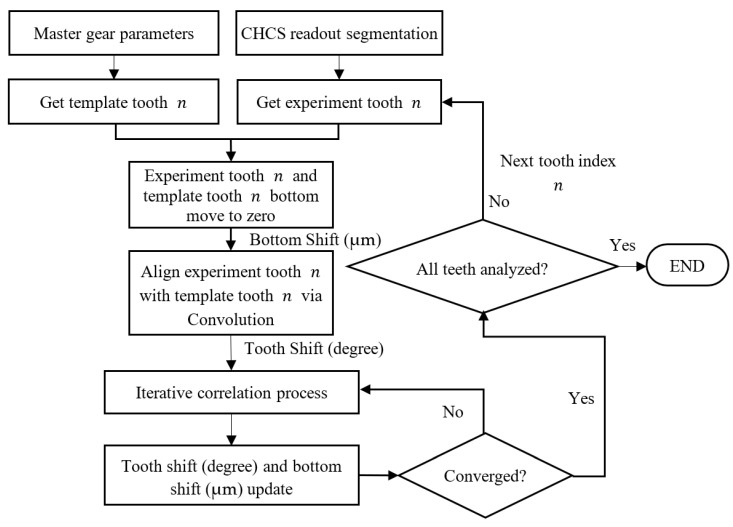
Flow chart of gear-center offset analysis.

The gear is not tightly mounted to the axle to improve the ease of the testing–gear exchange. The clearance preserved for the gear exchange, which can induce the experiment error due to gear-center offset. When the gear is mounted onto the motor servo-control system, the weight of the gear stays itself in the position of the motor axle with an unknown gear-center offset. The starting angle of the gear profile is not known in the CHCS readout when it is compared with the master gear template. As shown in Figure 12, we first moved both the gear tooth template to align the top-land and the experiment segmented tooth to the zero-CHCS-distance range and recorded their movement as the CHCS-readout bottom shift. We then apply the mathematical convolution calculation to shift the experiment tooth in order to cope with the template tooth for comparison. The micro-tuning of the tooth shift in degrees and the bottom shift in micrometers were subjected to the correlation process, which was achieved using iteration. The final tooth shift and the bottom shift are were recorded for each individual tooth, that is, how the tooth-left and tooth-right profiles can be compared in Figure 9 and Figure 10. It seems that when the gear teeth are whole on the gear, there should not be any difference in the tooth shift; however, according to Equation (27), when substituting into Equation (20), the relation between the motor angle θ and gear profile angle  ϕ deviates on each tooth.

**Figure 13:**
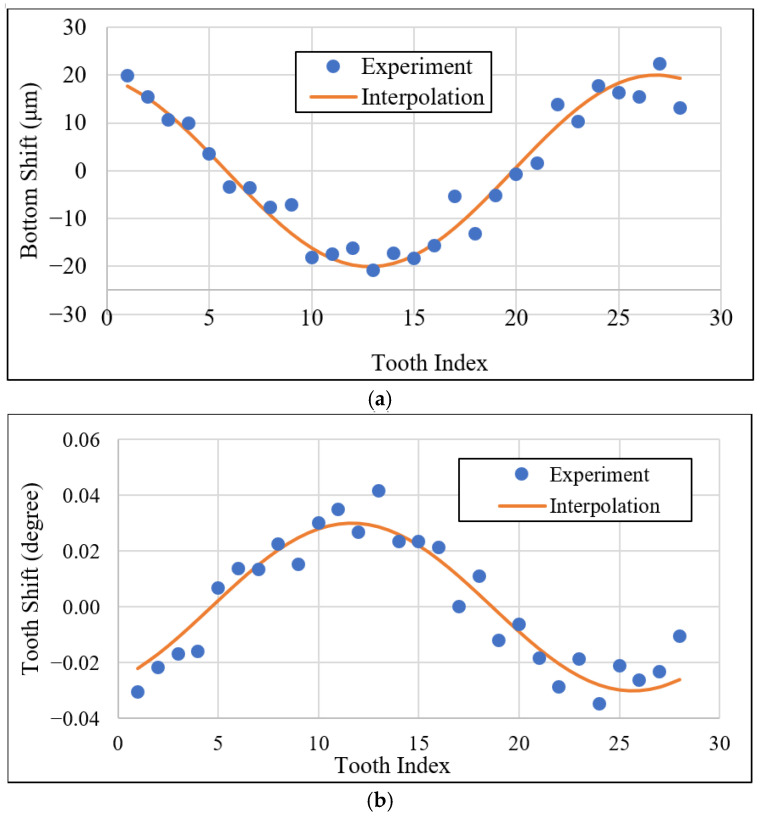
Results of gear-center offset analysis, including (**a**) bottom shift and (**b**) tooth shift.

Removing the common bottom and tooth shifts, we obtained the experimental result shown in Figure 13. According to the experimental result and using the cosine interpolation, $C_{1}\cdot cos(\frac{n}{N}\cdot 360+C_{2})$, we interpolated these data points in the experimental results for a full cycle to determine the amplitude $C_{1}$ and phase shift $C_{2}$. In Figure 13a, the experiment of “20.2C_Left” yielded the bottom shift interpolation as $20µm\cdot cos(\frac{n}{N}\cdot 360+15^{{}^{\circ}})$. According to Equation (29), we calculated the gear center offset set as 13 µm. The tooth indexed as tooth 13 in the experiment shall be the starting tooth, n=1, in the template, as shown in Figure 6. The tooth shift in degrees is also affected by the gear center-offset according to Equation (27) when substituting it into Equation (20). As shown in Figure 13b, the tooth shift due to the gear-center offset in degree interpolation is $0.04^{{}^{\circ}}\cdot cos(\frac{n}{N}\cdot 360+20^{{}^{\circ}})$. The maximum tooth shift is exhibited by the same tooth as the tooth presenting the maximum bottom-shift.

**Figure 14:**
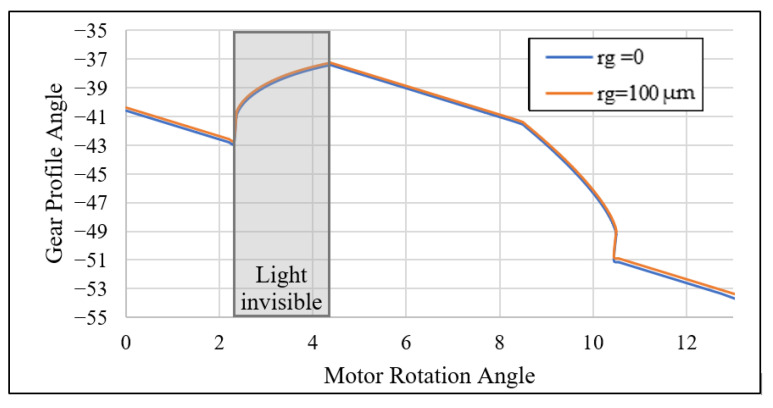
Gear profile angle related differently to the motor rotation angle via gear-center offset.

**Figure 15:**
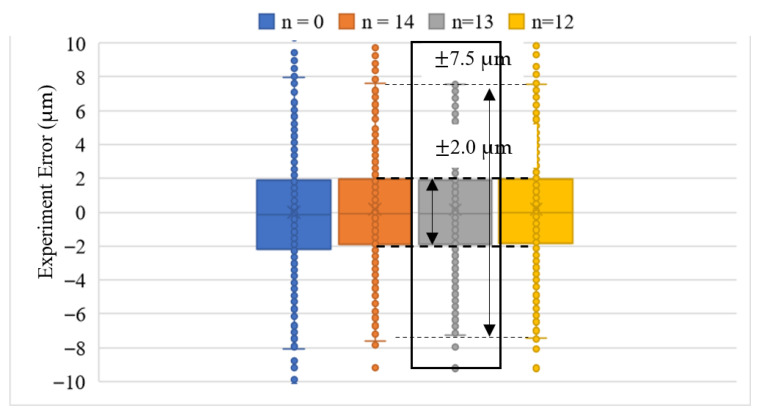
Error improvement by gear-center offset information feedback.

Since the experiment error occurred when comparing the experimental data with the standard template without the gear center-offset, as shown in Figure 14, the gear profile angles ϕ in terms of the motor rotation angle θ according to Equation (20) were different for zero and non-zero gear center-offset. The experiment error comparison result is also different when the gear-center offset is considered. We fed the gear-center offset information back into generating the standard template of individual teeth, which is in the form of Figure 7; the tooth profile of the standard template will be tooth-index-dependent. We compared the starting tooth of the gear with different tooth indices of the experiment with the experiment set “20.2C_Left” as an example. It was calculated from Figure 13 in the previous section that the tooth index n=13 should be recommended as the starting tooth defined in Figure 6. As shown in Figure 15, the best box-and-whisker plot result was obtained when we set the tooth index n=13 from the experiment as the starting tooth of the gear profile. With feedback from the gear-center-offset information, the quartile bounds can be further reduced from ±2.5 µm to ±2.0 µm, and the whisker bounds from ±8.0 µm to ±7.5 µm. The measurement resolution of gear products classified as acceptable (OK) or non-conforming (NG) was improved by 5%.

### 4.3. Precision Tolerance Specification for Gear Under Test

The gear profile evaluation parameters defined with reference to existing gear standards (e.g., ISO, DIN, and AGMA) always include gear radius, tooth run-out, tooth profile angle error, total tooth profile error, tooth profile shape error, average tooth profile pressure angle error, and V value. The threshold values, for example, the total tooth profile error, is 4 μm for 4th-grade spur gears in the DIN standard [9]. In this study, we established the 3D surface point retrieval method using CHCS; these 3D points can be used to calculate these errors, however, this is not the focus of this study.

In this section, instead of manufacturing multiple main gears for different gear specifications, we simulated various errors caused by tooth profile shape errors (including gear radius error, pressure angle error, and module error) according to gear standards (DIN, ISO, and AGMA), generating different gear profile templates, and comparing these templates with the original main gear. If the generated error gear profile template is considered correct, then the main gear is a non-conforming (or NG) part relative to these considered-correct templates. This allows us to determine the sensitivity of our precision tolerance specifications.

**Figure 16:**
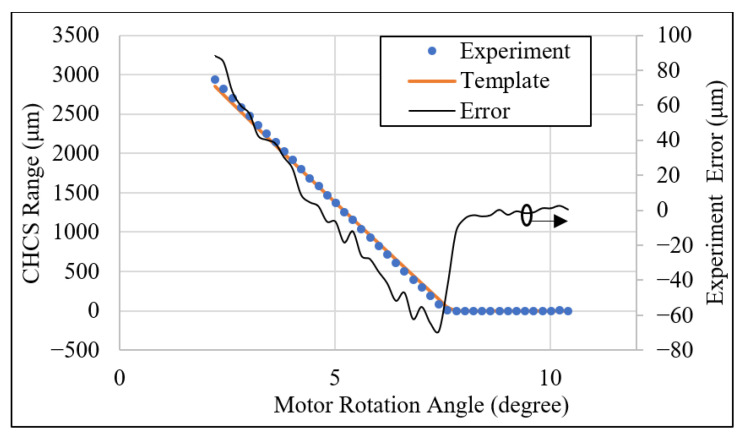
Tooth comparison between a GUT with a $15^{{}^{\circ}}$ pressure angle and the standard template.

**Figure 17:**
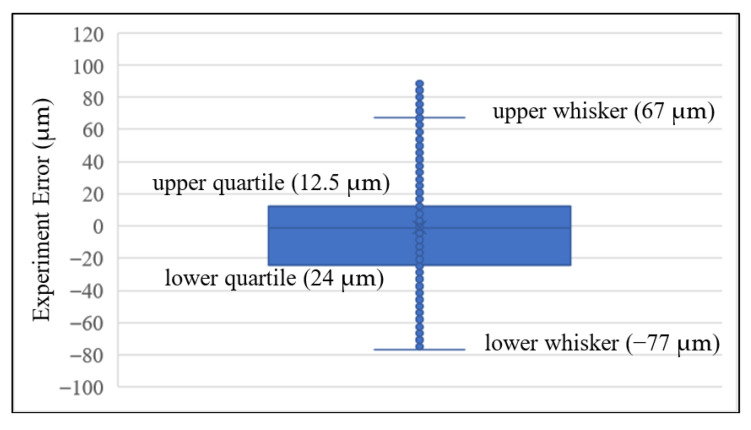
Box-and-whisker plot of the GUT result.

**Figure 18:**
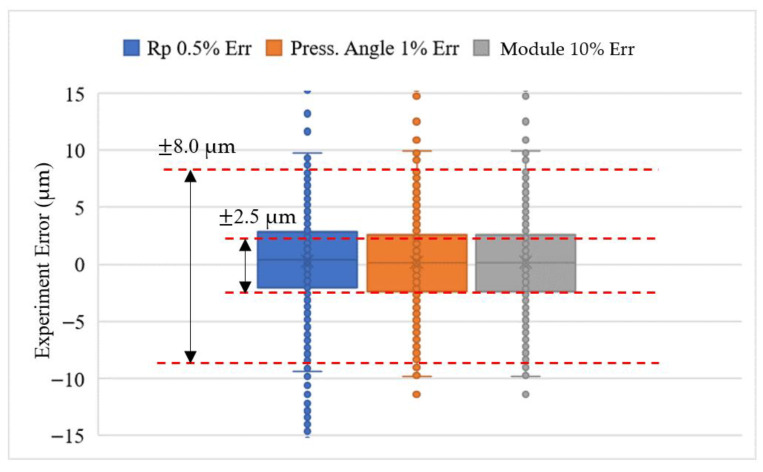
The precision tolerance of the GUT for passing the rapid profile inspection.

We used the gear parameters as listed in Table 1, except with a pressure angle of $15^{{}^{\circ}}$ against the master gear with a pressure angle remaining at $20^{{}^{\circ}}$, and going through the process flow as shown in Figure 12, we could compare one of the teeth as shown in Figure 16. The different pressure angles cause different involute profiles, which induce errors that both the convolution and least-square correlation processes were not able to compensate for. This finding enabled a rapid gear screening or sorting method that utilizes the box-and-whisker plot analysis as shown in Figure 17. Box plots show the five-number summary of a set of data, including the lower whisker, lower quartile, median, upper quartile, and upper whisker. As shown in Figure 18, the GUT has a quartile value beyond 2.5 μm and the whisker values are beyond 10 μm bounds, which may be deemed as the failure product compared with the gear parameters as listed in Table 1, except with a pressure angle of $15^{{}^{\circ}}$, if it is in a production line. The interpretation of the five numbers of the box-and-whisker plot analysis can imply the following error causes.

1. Central tendency: The line inside the box represents the median. A higher median indicates that the GUT profile is larger than the target gear, which needs to be finished more. A lower median indicates that the GUT profile is smaller than the target gear, which may be worn out.
2. Spread and variability: The length of the box represents the interquartile range (IQR), showing the spread of the middle 50% of the data. Smaller boxes indicate better precision, and vice versa.
3. Outlier identification: The whiskers typically extend to the minimum and maximum values within 1.5 times the IQR. Any points beyond the whiskers are considered either the heavier gear tooth modification, such as tip relief, or the collision damage on the gear tip.

Validating the box-and-whisker plot analysis, we tested the variation on the pitch circle radius error ($R_{p}$), the pressure angle error (α) and the module error (m) deviated from the gear parameters in Table 1. The same experiment data as shown in Figure 11, 20.2C_Left, are compared with a master gear with a 0.5% pitch circle radius error, 1% pressure angle error, and 10% module error, as shown in Figure 18. The median, i.e., central tendency, can increase due to the pitch circle increase. The spread and variability associated with the quartile change can result from the module error or the pressure angle differences. The outlier identification with whisker change can result from all errors. The box-and-whisker plot analysis of the GUT can relate to different error sources. Whenever the GUT violates the required bounds and median of the specification, it is deemed as a failure gear.

### 4.4. Used Gear Inspection

**Figure 19:**
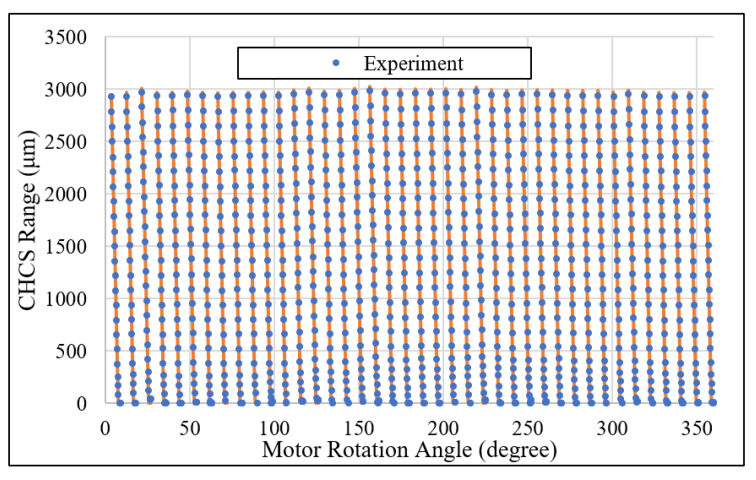
Tooth-left CHCS scan of the GUT with the gear specifications in Table 2.

**Figure 20:**
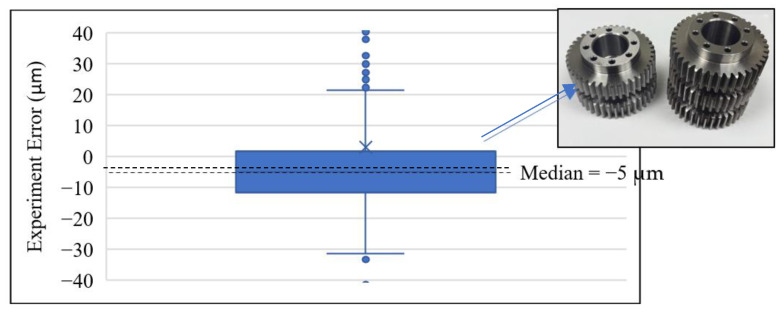
Box-and-whisker plot of a used gear.

A used gear, specified as shown in Table 2, is also included in the validation of the proposed gear inspection method. It helped us to understand the changes in the gear profile. The CHCS point mapping between the motor angle and the gear angles is again determined according to Equations (20) and (21), as shown in Figure 19. The experiment errors of the tooth-left profile are calculated between the CHCS readout and the template discrete position according to the motor rotation angle reading θ from the motor controller. In the tooth-left scan, we set $o_{L,y}=-28.698mm$. As shown in Figure 20, the box-and-whisker plot analysis of the error shows not only that the quartile values are beyond 2.5 μm, but also that the whisker values beyond 10 μm bounds as expected. This is due to the gear meshing collision, which refers to the impacts that occur when gear teeth make contact, primarily caused by system backlash (clearance) and external loads. The wearing condition also caused the median value of −5 μm according to the box-and-whisker plot analysis, which is a negative value. That is, the used gear is actually smaller than the template 40-teeth gear. The wearing conditions can result in stronger collision, generating vibrations and noise in geared system dynamics. 

## 5. Discussion

**Figure 21:**
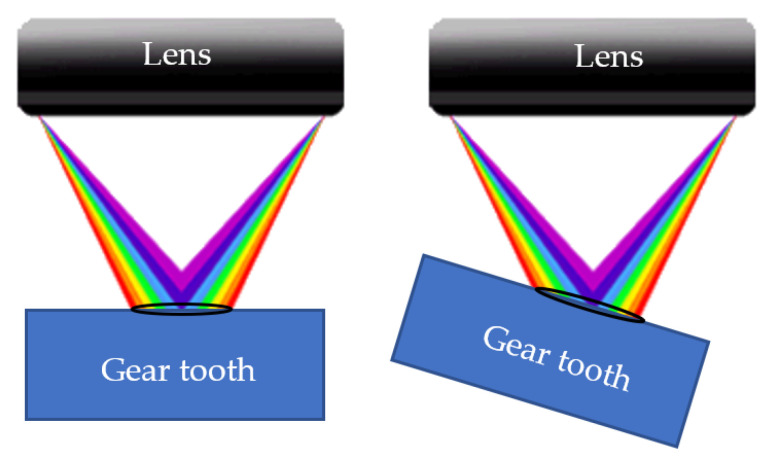
Light spot of CHCS.

A CHCS is an optical device that uses white and scattered light to measure distance. It achieves this by analyzing the reflection spectrum of a light spot on an object’s surface and correlating it with a specific wavelength. As shown in Figure 21, a 6 mm spot size does indeed yield a clearer reflection spectrum, allowing for better determination of whether the object is perpendicular or to the side, and more accurate calculation of the distance from the spot center to the lens. Discontinuities in the reflection spectrum indicate that light is illuminating multiple surfaces, resulting in an error message output. For CHCS manufacturers, there is always a trade-off between accuracy and resolution due to different spot sizes.

**Figure 22:**
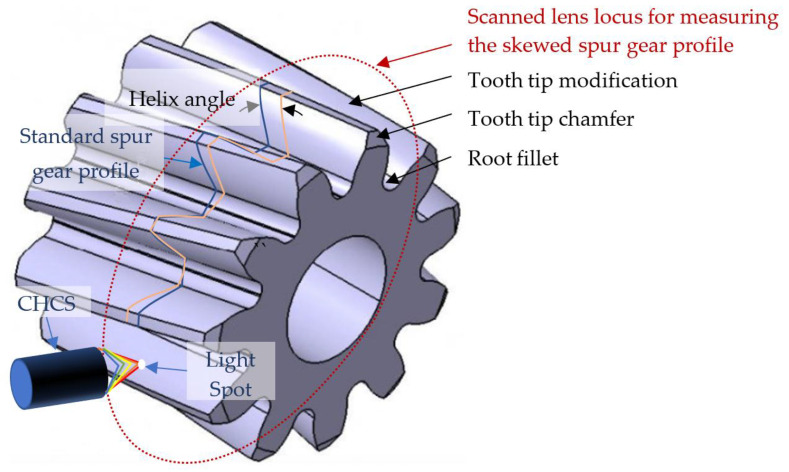
3D CAD model of a gear with gear modification.

This study proposes a method using CHCS for scanning a specific cross-sectional profile of a gear each time and determining the profile 3D positions. Therefore, a helical gear composed of multiple discrete involute spur gear cross-sections skewed along the helical axis may require multiple scans. Similar to modified gears, we need to develop software to compare each skewed cross-section with a 3D CAD model, as shown in Figure 22, to plot the deviation of each cross-section from the nominal profile. However, there is a boundary between online and offline measurements, requiring a trade-off between efficiency and accuracy.

In industrial applications, a CHCS sensor, an XY precision stage, and a computer are required. The XY stage moves the CHCS sensor to the side of the GUT and focuses it within its measurement range, which in this study is 53 mm from the gear tooth tip. Then, we start the spindle of a gear-processing machine (e.g., a gear grinder or gear-milling machine), select the cross-section of the gear for scanning, and convert the distance data $l_{\theta }$ into the gear profile F(ϕ) according to the formula presented in Section 2 (mainly Equation (22)). Error analysis is performed by comparing the measured gear profile F(ϕ) with the template generated by Equation (17) after removing the gear center offset error derived in Section 2.

The involute gear profile model in Formula (15) is intentionally defined only within the effective involute region, which begins at the involute generation point associated with the base circle and ends just before the theoretical tooth tip. The root fillet and tooth tip chamfer are not defined in the theoretical involute geometry; these gear corrections can be considered errors compared with the theoretical gear template derived from this formula. The tooth tip chamfer portion can be ignored when only the involute region is considered. Alternatively, gear modification errors can be analyzed by comparing the 3D CAD models shown in Figure 22 that are accurately modeled on the root fillet and tooth tip chamfer.

In this study, the eccentricity model described in Section 3.2 was specifically constructed for extremely small center offsets, indicating that, under high cutting forces, the elastic deformation of the chuck and spindle shaft will produce certain manufacturing errors. From a practical manufacturing perspective, once the eccentricity reaches a certain level (typically 2 to 3 μm), it has significant quantitative meaning relative to the module or tooth pitch, and the workpiece will fail to meet basic functional or inspection requirements and will be judged as a defective product. When this limit (2 to 3 μm) is exceeded, systematic biases may occur, including: nonlinear distortion of tooth profile representation, coupling between eccentricity and higher-order geometric errors, and mistaking deviations caused by eccentricity for true profile or pitch errors.

Before measurement, it is important to remove oil films and contaminants from the gears. Common methods include using industrial degreasers or solvent cleaners. In this study, we applied alcohol to the gear profile surface and blew the surface with an air gun to ensure the cleanliness standards of the gear manufacturer, thus guaranteeing measurement accuracy.

The coefficient of thermal expansion (CTE) of the S45C steel master gear with a radius of 30 mm is 12 ppm/°C, resulting in a radial expansion of 0.35 μm, as shown on the gear profile. In addition, CHCS is an optical device that uses white light and dispersed light to obtain distance by analyzing the reflection spectrum and correlating it with specific wavelengths. According to the PRECITEC user menu, the lateral resolution may be converted into 0.5 μm/℃ when the working range is 6 mm. Accumulated thermal fluctuations can lead to measurement errors of up to 0.85 μm/℃. The bounds of the upper and lower quartiles within ±2.5 μm indeed contain measurement uncertainties.

## 6. Conclusions

The CHCS was applied to gear inspection, which is a smaller piece equipment compared with the GMI and is convenient to install on the side of gear-fabricating machines. In the CHCS feasibility study for gear inspection, an ideal template with the nominal gear geometry was used, which itself did not contain any manufacturing tolerances. This feasibility study aimed to establish and validate the basic measurement capabilities, resolution, and repeatability of the proposed inspection method; therefore, the evaluation of the GUT was very rigorous. In practical applications, manufacturing tolerances can also be incorporated into the template according to specific application requirements or relevant standards. Gear inspection is available with in situ measurement using the proposed method. The precision of the CHCS was tested in a non-clean room, which presented itself as an acceptable version of the gear-inspection tool. The in situ measurement relies on box-and-whisker plot analysis and is based on the statistics of the point-to-point comparisons on the gear profile. In order to increase the CHCS light visibility on the gear profile, the CHCS is offset with a proper distance from the spindle center. The CHCS light points to the gear profile in a fixed position and orientation, and the gear profile is scanned by the motor. The theoretical CHCS point value was derived on the motor angle for error comparison, which formed a virtual template and was compared with the experimental CHCS point value. The error resulting from the gear tooth modification yields a non-zero whisker value; therefore, we will need a master gear for calculating the effect of the gear-tooth modification. The error resulting from the machining precision and accuracy yields a non-zero quartile value; therefore, the master gear needs to be labeled with the standard level. According to the GUT and master gear CHCS measurement result, the box-and-whisker plot analysis can yield not only the pass and NG signal, but also the error sources of the GUT. According to the analysis, the corresponding resolution can then be generated. The prototype experimental setup with light-weight CHCS equipment and a XY table is being implemented in a future study at the Industrial Technology Research Institute (ITRI), Taiwan.

## Figures and Tables

**Table 1:** Standard spur gear used in the verification of the theory derivation.

Symbol	Description	Unit	Value
m	module	mm/mm	2
N	number of teeth		28
$R_{p}$	radius of pitch circle	mm	28
α	pressure angle	degree	20
$R_{t}$	radius of top circle	mm	29.351
Q	number of interpolation points		100
$q_{0}$	starting parameter q		0
$R_{de}$	radius of dedendum circle	mm	25.5
$q_{t}$	Equation (9)		124.45
$\phi_{0}$	Equation (12)	deg	2.3603
$\phi_{p}$	Equation (7)	deg	3.2143
$\phi_{t}$	Equation (13)	deg	4.3792
$\phi_{1}$	Equation (11)	deg	6.4286
$\phi_{tl}$	Equation (14)	deg	4.0989

**Table 2:** Gear specification of a 40-teeth spur gear.

Symbol	Description	Unit	Value
m	module	mm/mm	2
N	number of teeth		40
$R_{p}$	radius of pitch circle	mm	40
α	pressure angle	degree	20
$R_{t}$	radius of top circle	mm	41.974
$q_{0}$	starting parameter q		0
$R_{de}$	radius of dedendum circle	mm	37.5
$o_{L,x}$	CHCS position x	deg	33.929
$o_{L,y}$	CHCS position y	deg	28.698

## Data Availability

The original contributions presented in this study are included in the article. Further inquiries can be directed to the corresponding author.

## Abbreviations

AR6	Sixth Assessment Report
CCDs	Charge-Coupled Devices
CCW	Counterclockwise
CHCSs	Chromatic Confocal Sensors
CMM	Coordinate Measuring Machine
CTE	Coefficient of Thermal Expansion
CW	Clockwise
DFT	Double Flank Test
GMI	Gear-Measuring Instrument
GUT	Gear Under Test
IPCC	Intergovernmental Panel on Climate Change
IQR	Interquartile Range
ITRI	Industrial Technology Research Institute
LED	Light-Emitting Diode
m	Module
N	Number of Teeth
NG	Non-Conforming
OK	Acceptable
$q_{0}$	Starting Parameter q
$R_{de}$	Radius of Dedendum Circle
$R_{p}$	Radius of Pitch Circle
$R_{t}$	Radius of Top Circle
SFT	Single Flank Test
α	Pressure Angle

## References

[1] Shao T., Pan X., Li X., Zhou S., Zhang S., Chen W. (2022). China’s industrial decarbonization in the context of carbon neutrality: A sub-sectoral analysis based on integrated modelling. Renew. Sustain. Energy Rev..

[2] Pai W., Liang J., Zhang M.-K., Tang Z., Li L. (2022). An advanced multi-camera system for automatic, high-precision and efficient tube profile measurement. Opt. Lasers Eng..

[3] Guan B., Zhao J., Mitra S., Kneip L. (2025). Six-point method for multi-camera systems with reduced solution space. Int. J. Comput. Vis..

[4] Pueo M., Santolaria J., Acero R., Gracia A. (2017). A review of tangential composite and radial composite gear inspection. Precis. Eng..

[5] Tang J., Jiang K.J., Wu X.H. (2025). Measurement method of cylindrical spur gears on CMM and its software development. J. Beijing Univ. Technol..

[6] Goch G. (2003). Gear metrology. CIRP Ann. Manuf. Technol..

[7] (2013). Cylindrical Gears—ISO System of Flank Tolerance Classification—Part 1: Definitions and Allowable Values.

[8] (2008). Cylindrical Gears—Accuracy—Part 1: Definitions and Allowable Values.

[9] (1978). Accuracy of Cylindrical Gears—Tolerances and Deviations.

[10] (1988). Gear Classification and Inspection Handbook—Tolerances and Measuring Methods for Unassembled Spur and Helical Gears.

[11] Smith R.E. (1984). Single-flank testing of gears. Gear Technol..

[12] Reiter E., Eberle F. (2014). Practical considerations for the use of double-flank testing for the manufacturing control of gearing—Part I. Gear Technol..

[13] Reiter E., Eberle F. (2014). Practical considerations for the use of double-flank testing for the manufacturing control of gearing—Part II. Gear Technol..

[14] Wang X., Liu M., Yao T., Zheng K., Zhao C., Xiao L., Zhu D. (2023). Study on the measurability of gear analytical parameters in double-flank measurement. Sensors.

[15] Baldwin J.M., Summerhays K.D., Campbell D.A., Henke R.P. (2004). Application of Simulation Software to Coordinate Measurement Uncertainty Evaluation.

[16] Cui C., Li X. (2025). Precision Nanometrology: Laser Interferometer, Grating Interferometer and Time Grating Sensor. Sensors.

[17] Van Gestel N., Cuypers S., Bleys P., Kruth J.-P. (2009). A performance evaluation test for laser line scanners on CMMs. Opt. Lasers Eng..

[18] Younes M.A., Khalil A.M., Damir M.N. (2005). Automatic measurement of spur-gear dimensions using laser light. Part 1: Measurement of tooth thickness and pitch. Opt. Eng..

[19] Younes M.A., Khalil A.M., Damir M.N. (2005). Automatic measurement of spur gear dimensions using laser light, Part 2: Measurement of flank profile. Opt. Eng..

[20] (2008). Evaluation of Measurement Data—Guide to the Expression of Uncertainty in Measurement (GUM 1995 with Minor Corrections).

[21] Auerswald M.M., von Freyberg A., Fischer A. (2024). Laser line triangulation for fast 3D measurements on large gears. Int. J. Adv. Manuf. Technol..

[22] Li C., Li K., Liu J., Lv Z., Li G., Luo D. (2023). Design of dispersive objective lens for spectral confocal displacement sensor based on GRIN lens. Proc. SPIE.

[23] Li J., Ma R., Bai J. (2024). High-Precision Chromatic Confocal Technologies: A Review. Micromachines.

[24] He N., Hu H., Cui Z., Xu X., Zhou D., Chen Y., Gong P., Chen Y., Kuang C. (2024). Compact Chromatic Confocal Lens with Large Measurement Range. Sensors.

[25] Pillarz M., von Freyberg A., Fischer A. (2020). Gear shape parameter measurement using a model-based scanning multi-distance measurement approach. Sensors.

[26] Pillarz M., von Freyberg A., Fischer A. (2020). Determination of the mean base circle radius of gears by optical multi-distance measurements. J. Sens. Sens. Syst..

[27] Precitec Optronik GmbH How Chromatic Confocal Sensor Technology Works. https://www.precitec.com/optical-3d-metrology/technology/chromatic-confocal-sensors.

